# Ecological and environmental factors affecting transmission of sylvatic yellow fever in the 2017–2019 outbreak in the Atlantic Forest, Brazil

**DOI:** 10.1186/s13071-021-05143-0

**Published:** 2022-01-10

**Authors:** Filipe Vieira Santos de Abreu, Cecilia Siliansky de Andreazzi, Maycon Sebastião Alberto Santos Neves, Patrícia Soares Meneguete, Mário Sérgio Ribeiro, Cristina Maria Giordano Dias, Monique de Albuquerque Motta, Christovam Barcellos, Anselmo Rocha Romão, Mônica de Avelar Figueiredo Mafra Magalhães, Ricardo Lourenço-de-Oliveira

**Affiliations:** 1 Laboratório de Mosquitos Transmissores de Hematozoários, Instituto Oswaldo Cruz, FIOCRUZ, Rio de Janeiro, RJ Brazil; 2 Laboratório de Comportamento de Insetos, Instituto Federal do Norte de Minas Gerais, Salinas, MG Brazil; 3 Laboratório de Biologia e Parasitologia de Mamíferos Silvestres Reservatórios, Instituto Oswaldo Cruz, FIOCRUZ, Rio de Janeiro, RJ Brazil; 4 Present Address: Centre for Functional Ecology, Department of Life Sciences, University of Coimbra, Calçada Martim de Freitas, 3000-456 Coimbra, Portugal; 5 Secretaria de Estado de Saúde, Subsecretaria de Vigilância e Atenção Primária À Saúde, Rio de Janeiro, RJ Brazil; 6 Laboratório de Informação em Saúde, Instituto de Comunicação e Informação Científica e Tecnológica em Saúde, FIOCRUZ, Rio de Janeiro, RJ Brazil

**Keywords:** *Haemagogus*, Mosquito, Nonhuman primate, Functional traits

## Abstract

**Background:**

Yellow fever virus (YFV) is an arbovirus that, despite the existence of a safe and effective vaccine, continues to cause outbreaks of varying dimensions in the Americas and Africa. Between 2017 and 2019, Brazil registered un unprecedented sylvatic YFV outbreak whose severity was the result of its spread into zones of the Atlantic Forest with no signals of viral circulation for nearly 80 years.

**Methods:**

To investigate the influence of climatic, environmental, and ecological factors governing the dispersion and force of infection of YFV in a naïve area such as the landscape mosaic of Rio de Janeiro (RJ), we combined the analyses of a large set of data including entomological sampling performed before and during the 2017–2019 outbreak, with the geolocation of human and nonhuman primates (NHP) and mosquito infections.

**Results:**

A greater abundance of *Haemagogus* mosquitoes combined with lower richness and diversity of mosquito fauna increased the probability of finding a YFV-infected mosquito. Furthermore, the analysis of functional traits showed that certain functional groups, composed mainly of Aedini mosquitoes which includes *Aedes* and *Haemagogus* mosquitoes, are also more representative in areas where infected mosquitoes were found. Human and NHP infections were more common in two types of landscapes: large and continuous forest, capable of harboring many YFV hosts, and patches of small forest fragments, where environmental imbalance can lead to a greater density of the primary vectors and high human exposure. In both, we show that most human infections (~ 62%) occurred within an 11-km radius of the finding of an infected NHP, which is in line with the flight range of the primary vectors.

**Conclusions:**

Together, our data suggest that entomological data and landscape composition analyses may help to predict areas permissive to yellow fever outbreaks, allowing protective measures to be taken to avoid human cases.

**Graphical Abstract:**

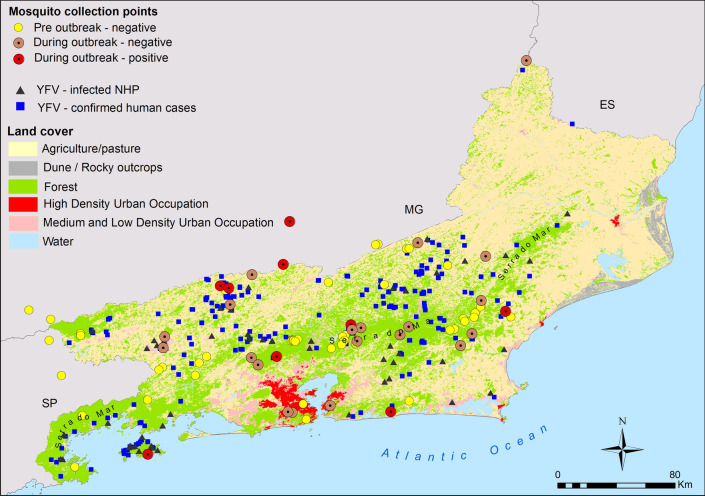

**Supplementary Information:**

The online version contains supplementary material available at 10.1186/s13071-021-05143-0.

## Background

Yellow fever is an important arboviral disease characterized by febrile and acute symptoms, with high mortality rates. Despite the existence of a safe and effective vaccine since the 1930s, yellow fever virus (YFV) continues to cause outbreaks of varying dimensions in the Americas and Africa [1].

In Brazil, the urban cycle of yellow fever, in which YFV is transmitted between humans through the bite of the domestic mosquito *Aedes aegypti*, has not been recorded since the early 1940s. However, the sylvatic cycle, where transmission between nonhuman primates (NHPs) occurs by the bite of arboreal mosquitoes, remains active in Brazil and other South American countries [2]. This YFV sylvatic cycle produces epizootic waves of expansion followed by retractions, threatening NHP populations at risk of extinction and affecting humans who live in the border of epizootic forests or who visit them for leisure or work [2–4]. The enzootic sylvatic transmission cycle seems to be perennial in the tropical rainforest of northern South America, particularly in the Amazon, from which YFV can initiate epizootic expansion waves affecting other biomes. In Brazil, the YFV expansion waves have spread towards the south and east, where the human population density is much higher than in the Amazon but vaccination coverage is frequently lower [2, 5]. The last YFV epizootic wave started in 2014/2015 in the Amazon and is still active today (2021) in Southern Brazil [6]. It is the largest recorded YFV outbreak. Between 2015 and 2019, YFV crossed the entire southeastern region of Brazil, where the largest and most populous cities infested by *Ae. aegypti* are located, raising concerns about the risk of re-urbanization of YFV transmission [2, 7–10].

The virus has also spread to the coastal region of Brazil covered by the Atlantic Forest that had been considered a YFV-free area for the last 80 years and whose human population therefore had even lower vaccination coverage. This was the case of the state of Rio de Janeiro (RJ), where YFV had not been detected since the late 1930s [2, 11]. The state was severely affected during the passage of the 2017–2019 epizootic wave, thus constituting an important place to study the factors involved in the YFV re-emergence. The YFV wave reached RJ in February 2017, entering from its northern portion, which borders Espírito Santo, a state that had already registered YFV infections in humans and NHPs since January 2017 [12, 13]. The virus circulated in several regions of RJ until the last detection in January 2019 [14]. In total, 27 and 262 human cases and 9 and 84 deaths were confirmed for RJ in 2017 and 2018, respectively [15]. In 2019, there was only one confirmed NHP infection with YFV in RJ [14]. Genetic and molecular studies of YFV samples demonstrated that the virus spread from the northeast to southwest of RJ through two different transmission routes, one coastal and one continental, separated by the Serra do Mar mountain chain [9, 13]. Systematic mosquito collections performed during the outbreak showed that the primary mosquito vectors in RJ and other states in southeastern Brazil were *Haemagogus janthinomys*/*capricornii* and *Hg. leucocelaenus.* Three other mosquito species (*Sabethes chloropterus*, *Aedes scapularis*, and *Ae. taeniorhynchus*) were found infected with YFV, but were considered to have a secondary role in transmission in this outbreak [16]. Concerning NHPs, 1177 deaths were recorded in RJ. Howler monkeys (*Alouatta guariba clamitans*) were proportionally the most highly affected species and those with the highest viral load [17, 18]. Although a considerable amount of information was available on the epidemiological aspects concerning humans and monkeys, the vectors, the virus, and the routes traveled by it in RJ, understanding the ecological and environmental factors that increase the chances of local YFV occurrence and its infection rates is key to improving yellow fever surveillance and control measures.

Indeed, the influence of climatic, environmental, and ecological factors governing the distribution of YFV primary vectors, as well as the dispersion and force of infection of YFV in a naïve area such as RJ, is still poorly known. The increase in rainfall, relative humidity of the air, temperature, and the number of local NHP species have been identified as determining factors for the occurrence of yellow fever and used as elements for the prediction of yellow fever risk in other Brazilian regions [19–21]. In the present work we used a multidisciplinary approach combining geographical, ecological, and entomological data to investigate the factors affecting YFV transmission and to better understand the spread of YFV in the landscape mosaic of RJ during the 2017–2019 outbreak.

## Methods

### Mosquito sampling

Mosquito captures were made according to methods described elsewhere [16]. Briefly, expeditions were performed between 2015 and 2019 (before and during YFV outbreak) in order to investigate fauna composition in different regions of RJ, which constitutes the country's second largest economy and the third largest population (6.75 million inhabitants), and is considered the main country gateway for foreign tourists [22]. Adult mosquitoes were captured through hand nets, mechanical aspirators, and CO_2_-baited BG-traps, frozen in liquid nitrogen, and subsequently tested by polymerase chain reaction (PCR) for YFV detection [16]. Each mosquito sampling point was georeferenced and classified according to the predominant environment (inside the forest—within dense forests connected to other forests; rural fragment—within forests smaller than 100 ha and surrounded by pastures; rural peri-domicile—around homesteads and country houses; urban fragment—within forests inside cities; and urban intra-domicile—within human houses inside cities) for ecological analyses. Geo-environmental characteristics including altitude, land cover/land use, forest fragment size, and Normalized Difference Vegetation Index (NDVI) [23] were also measured for each sampling point. Collected mosquitoes were classified up to the lowest taxonomic level using dichotomous keys [24–26], and data were tabulated according to the sampling point, date, and effort of capture (expressed in numbers of hours, traps, and people working in the capture).

#### Mosquito functional traits

To analyze the functional diversity of mosquito species at each sampling point, we selected nine categorical parameters (Table 1) related to behavior, physiology, habitats, and epidemiological importance, as follows: 1—oviposition preferences; 2—egg resistance to desiccation; 3—larval development speed; 4—host preference; 5—main hourly biting activity; 6—vertical distribution in the forest; 7—seasonal distribution; 8—main habitat; 9—epidemiological importance concerning YFV. We built a traits × species matrix, and the functional distances between pairs of species were computed using the Gower distance [27]. Functional groups were identified based on a functional dendrogram using the Ward hierarchical agglomerative clustering method [28]. Principal components analysis (PCA) was performed using the package FactoMineR [29] to summarize the relationships among traits and identify those most commonly shared among species from the same functional group.

**Table 1:** List of functional traits used for each collected mosquito species

Functional trait	Description	Functional meaning	References
Larval habitat/oviposition behavior	(1) Artificial containers; (2) natural containers; (3) natural groundwater—shallow; (4) natural groundwater—deep; (5) tree hollows; (6) perforated bamboo; (7) cut bamboo; (8) skip oviposition	Drives survival and dispersion of the offspring; inter- and intraspecific competition during larval development; environmental requirements for specific breeding sites; domiciliation capacity	[24, 50, 72–81]
Egg resistance to desiccation	Resistant; nonresistant	Indicates the ability to wait for favorable conditions for immature development	[24, 48, 72–74]
Larval development speed	Fast (up to 10 days); slow (more than 10 days)	Determines the immature survival (or not) in temporary breeding sites; sudden population increases	[24, 72, 78–81]
Host preference	Primatophilic; ornithophilic; eclectic/opportunistic	Related to the probability of transmitting certain pathogens between host groups (e.g., YFV transmission to human and nonhuman primates by primatophilic mosquitoes), and interspecific competition	[24, 55, 82–85]
Main hourly biting activity	Diurnal; nocturnal; twilight/eclectic	Reflects the period of activity, the behavior of mosquitoes, and the finding of hosts and interspecific competition	[24, 26, 78, 86, 87]
Vertical distribution in the forest	Mostly on the ground level; mostly at the tree canopy; eclectic/opportunistic (canopy and ground)	Determines both breeding sites and exploited hosts. Eclectic mosquitoes can serve as bridge vectors of pathogens from canopy-dwelling hosts to ground-dwelling hosts and vice versa	[88–94]
Seasonal distribution	Accentuated (abrupt population peaks); moderate (no abrupt peaks)	Populations increase abruptly in response to certain environmental events (e.g., rain, temperature), which increases vectorial capacity and determines the most favorable periods for transmission of pathogens	[50, 60, 72, 78, 87, 92, 95–99]
Environment	Forest interior; forest edge; peri-urban; urban	Ability to withstand different degrees of environmental impact and ecological impoverishment. Also reflects anthropophily and interspecific competition	[46, 60, 83, 94, 98, 100]
Epidemiological importance	YFV natural infection; YFV transmission in experimental infections; YFV primary vector; YFV secondary or local vector	Indicates the accumulated evidence for vector competence and vectorial capacity of the species	[16, 43, 44, 101–112]

#### Geolocation of YFV infections in humans and NHPs

The geographical coordinates of the most probable local infections (PLI) of humans as well as places where YFV-infected NHPs were found dead were determined based on information obtained from the RJ State Department of Health or through investigations in each of the affected municipalities, supported by the local health departments. To observe the effect of the landscape on the YFV transmission, the coordinates were plotted on a map containing land use/land cover in RJ. In addition, kernel maps were generated by plotting the most probable place of YFV infections in humans, NHPs, and both to verify areas with higher infection forces. Radii of 5 and 11 km, consistent with the flight radius of *Haemagogus* mosquito species considered the primary vector during the outbreak [30], were plotted from each positive NHP to verify the minimum distance between an epizootic and the findings of human cases. Radii of 5 and 11 km were also plotted from the place where each YFV-positive mosquito was caught to verify the minimum distance between it and the PLI of human cases.

### Data analysis

#### Biodiversity analysis

We built a site × species mosquito abundance matrix containing data from the 84 sampling points and 89 mosquito species sampled. A square root transformation was applied to the raw mosquito species counts to dampen the effect of dominant species, and the transformed counts were then divided by the effort of capture to control for differing sampling efforts. Analyses were performed by comparing (1) the sampling carried out before the yellow fever epidemic with that conducted during the outbreak (the latter was subdivided into sampling points with infected and uninfected mosquitoes), (2) the sampling points sampled during the yellow fever epidemic, and (3) the total period sampled, as described below.

(ii) *Comparing the three different scenarios:* Differences in mosquito community composition among the three epidemiological scenarios (before YFV outbreak vs. during YFV outbreak positive points vs. during YFV outbreak negative points) were analyzed using a permutational multivariate analysis of variance (PERMANOVA) based on the Bray–Curtis distance matrix [31]. SIMPER (similarity percentage) analysis was applied to assess which species were primarily responsible for the observed differences. These analyses were conducted using the vegan package in R 4.0.3 [32].

Mosquito community diversity was characterized by two measures: (1) mosquito species richness and (2) the Shannon–Wiener index (Table 2) [33]. The functional diversity of mosquito communities was calculated on a continuous scale using four different indices: (1) functional richness (FRic) [34], (2) functional evenness (FEve) [34], (3) functional divergence (FDiv) [34], and (4) functional dispersion (FDis) (Table 2) [35]. The community-weighted means (CWM) of traits [36] were calculated for each sampling point by averaging the trait expression of all mosquito species weighted by their relative abundance. Variation in CWM trait composition among habitat categories was addressed by employing canonical correspondence analysis (CCA). For the CCA, we did not include species traits related to environment (Table 1) in the CWM matrix because we used the habitat descriptions from the sampled areas as environmental factors. Functional diversity analysis and CCA analysis were performed using the FD and vegan packages in R 4.0.3, respectively [32, 37].

**Table 2:** Variables analyzed in the present study

Variables	Definition
Species richness	Total number of species sampled at each sampling point
Shannon–Wiener index	Measure of species diversity weighted by relative abundance [33]
Functional richness (FRic)	Represents the quantity of functional space filled by the community, where low FRic implies that some resources are unused or unavailable in the ecosystem [34]
Functional evenness (FEve)	Describes the distribution of abundance in a functional space of traits, where low FEve indicates that some parts of the functional niche are underutilized [34]
Functional divergence (FDiv)	A measure of the functional similarity among the dominant mosquito species of a community. FDiv is high when the most abundant species have extreme functional trait values [34]
Functional dispersion (FDis)	A multivariate measure of the dispersion of mosquito species in the trait space, and represents the mean distance of species to the centroid of the community, weighted by mosquito species abundance [35]
*Haemagogus* relative abundance	The number of *Haemagogus* mosquitoes divided by the total number of mosquitoes collected at each sampling point
*Haemagogus* minimum infection rate (MIR)	Represents the minimum number of infected mosquitoes, assuming that in each positive mosquito pool only one was infected. It was calculated for each sampling point using the formula MIR = number of YFV-positive *Haemagogus* pools / total number of *Haemagogus* tested (only the points sampled during YFV outbreak were considered, as there was no evidence of YFV circulation in the past 70 years)
Positivity of sampling points	Binary variable. Equal to 1 when at least one mosquito pool tested positive for YFV (only the points sampled during YFV outbreak were considered)
Altitude	The altitude related to sea level for each sampling point
Normalized Difference Vegetation Index (NDVI)	Analyzes the conditions of the vegetation coverage through images generated by remote sensing [23]
Fragment size	The area of the forested patch around each sampling point
Land use/land cover	Categorical variable describing the type of human activity and vegetation cover at each sampling point

(ii) *Comparing only the sampling points sampled during the yellow fever epidemic (2017–2019)—infection predictors:* Generalized linear mixed models (GLMMs) were used to investigate the effects of mosquito biodiversity (richness, Shannon–Wiener index, FRic, FEve, FDiv, and FDis) and *Haemagogus* relative abundance on (1) the *Haemagogus* minimum infection rate (MIR) and (2) the positivity of sampling points (Table 2). For that, only the points sampled during YFV outbreak were considered. *Haemagogus* MIRs were related to the predictors by fitting zero-inflated models with negative binomial errors and log link functions, and the positivity of sampling points by fitting models with binomial errors and logit link functions. A model-averaging approach was applied, which accounts for model uncertainty, increases the robustness of the parameter estimates, and assesses the relative importance of each of the predictor variables [38, 39]. Model averaging started with a global model with all the predictor variables previously described, fitted using the glmmTMB package in R [40]. The dredge function of the MuMIn package [41] was used to create a set of models with all combinations of variables. The Akaike information criterion with correction for small sample size (AICc) [42] was used to identify the best models based on the averaged model including all the equally plausible models (ΔAICc ≤ 2). Averaged parameter estimates were calculated from this set of selected models. We calculated the relative importance of each variable using the model.avg function. The relative importance was calculated using the sum of Akaike weights across all the selected models, with a weight of zero for models where a given parameter was absent [38]. In addition, we calculated the McFadden index (*R*^*2*^*M*) of the selected models as a measure of model fit.

(ii) *Comparing the entire sampled period (2015–2019):* As *Hg. janthinomys*/*capricornii* and *Hg. leucocelaenus* are the primary vectors of yellow fever, Pearson correlation tests were carried out to verify whether geo-environmental characteristics (altitude, NDVI, fragment size, land use/land cover) were related to the relative abundance of these species.

## Results

### Biodiversity analysis

#### Comparisons among the three different scenarios

Mosquito community composition varied among the three epidemiological scenarios (PERMANOVA, pseudo-*F* = 1.3258, *P*-perm = 0.04096). *Aedes albopictus*, *Hg. leucocelaenus*, and *Hg. janthinomys*/*capricornii* were the species that most contributed to overall dissimilarity between positive and negative sampling points during YFV outbreak, together accounting for 28% of the differences among scenarios.

**Fig. 1:**
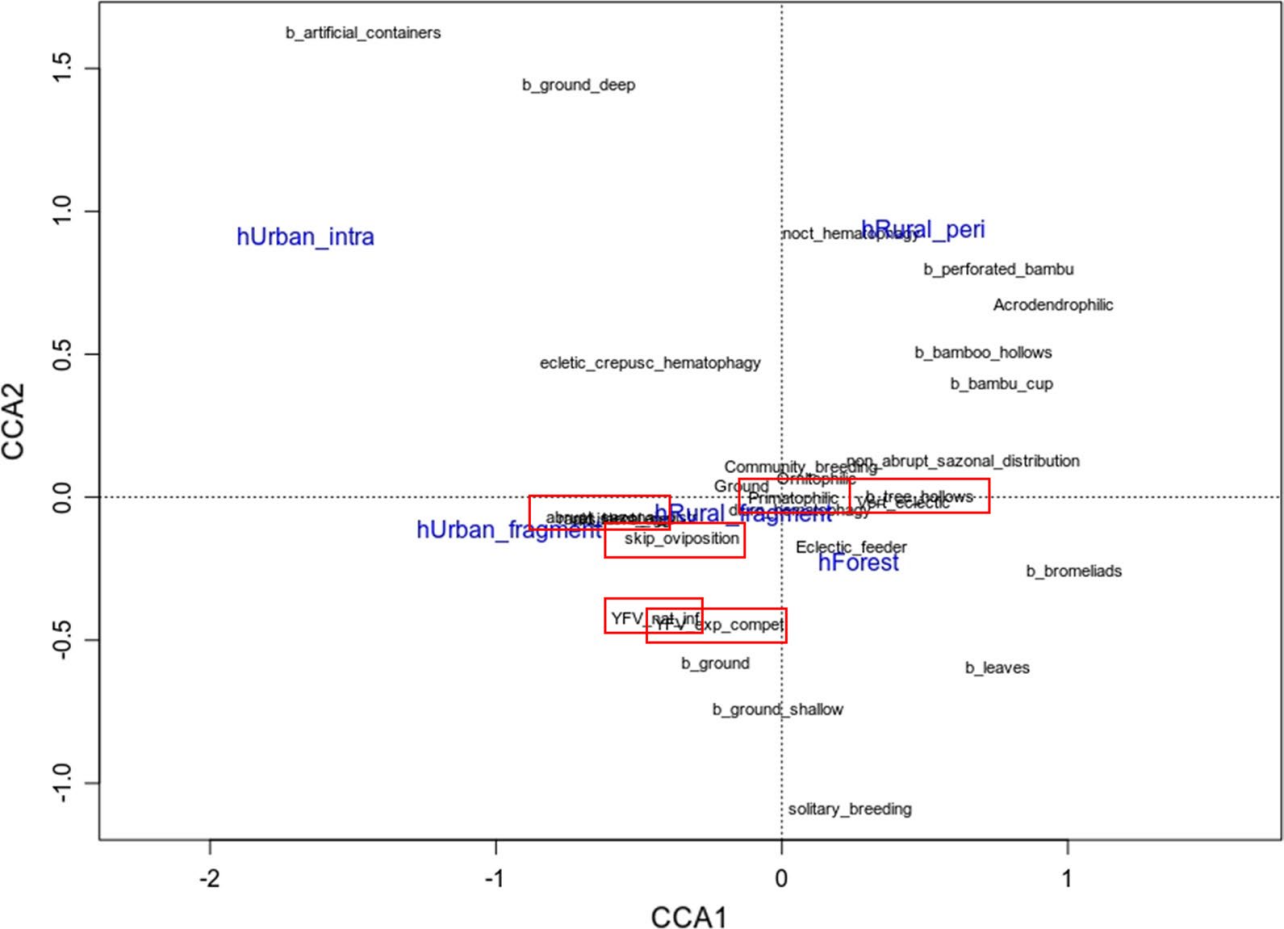
Canonical correspondence analysis (CCA) of mosquito trait community-weighted means (CWM) and habitat categories (forest, rural fragment, urban fragment, rural peri-domicile, and urban intra-domicile). Red rectangles highlight traits related to the main yellow fever vectors

The CCA results revealed that habitat categories (rural fragment, urban fragment, forest interior, urban intra-domicile, and rural peri-domicile) explained a significant amount of variation in mosquito community mean traits (CWM, ANOVA-like test, 999 permutations; *F* = 2.69, *P* = 0.001). The first and second CCA axis explained 30% and 17% of the variance, respectively, and together they accounted for 47% of the explained variation and 12% of total variation in the data (Fig. 1). In other words, traits related to the main yellow fever vectors (e.g. YFV natural infection, YFV experimental vector competence, primatophilic behavior, and skip oviposition) are more common in forest fragments (both rural and urban) and, secondarily, forested habitats (Fig. 1).

**Fig. 2:**
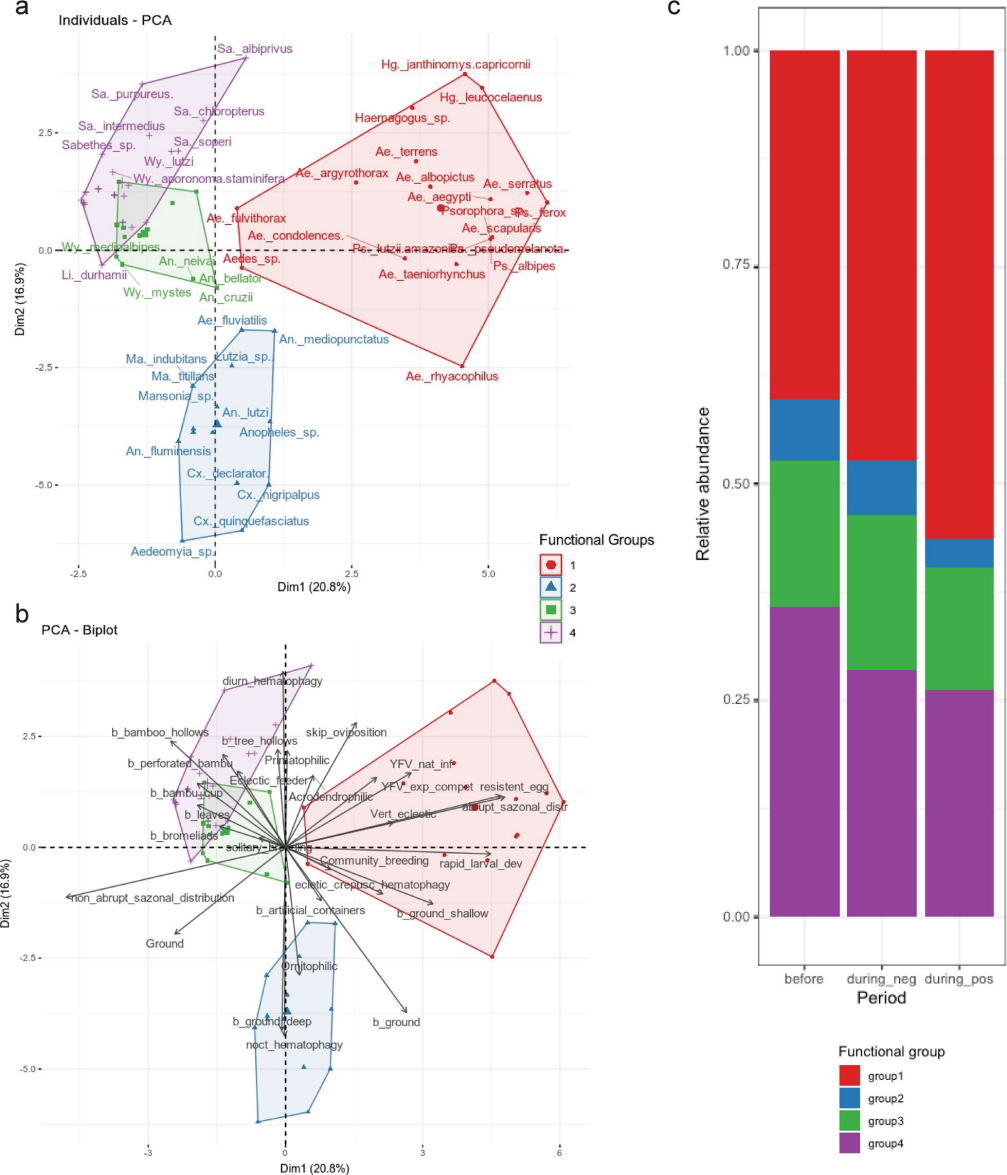
Functional groups formed through mosquito functional trait analysis. **a** Mosquito species forming each functional group. **b** Ecological traits forming each functional group. **c** Relative abundance of each functional group in the three epidemiological scenarios

The first two axes of the PCA explained 37.7% of the variance (Fig. 2). Altogether, we found four functional groups (Fig. 2), one of which—group 1—contained a concentration of most of the mosquitoes of tribe Aedini, including *Haemagogus*, primary vectors of yellow fever (Fig. 2a and b). Interestingly, the relative abundance of this functional group was increased at sampling points with YFV-positive mosquitoes (Fig. 2c).

**Fig. 3:**
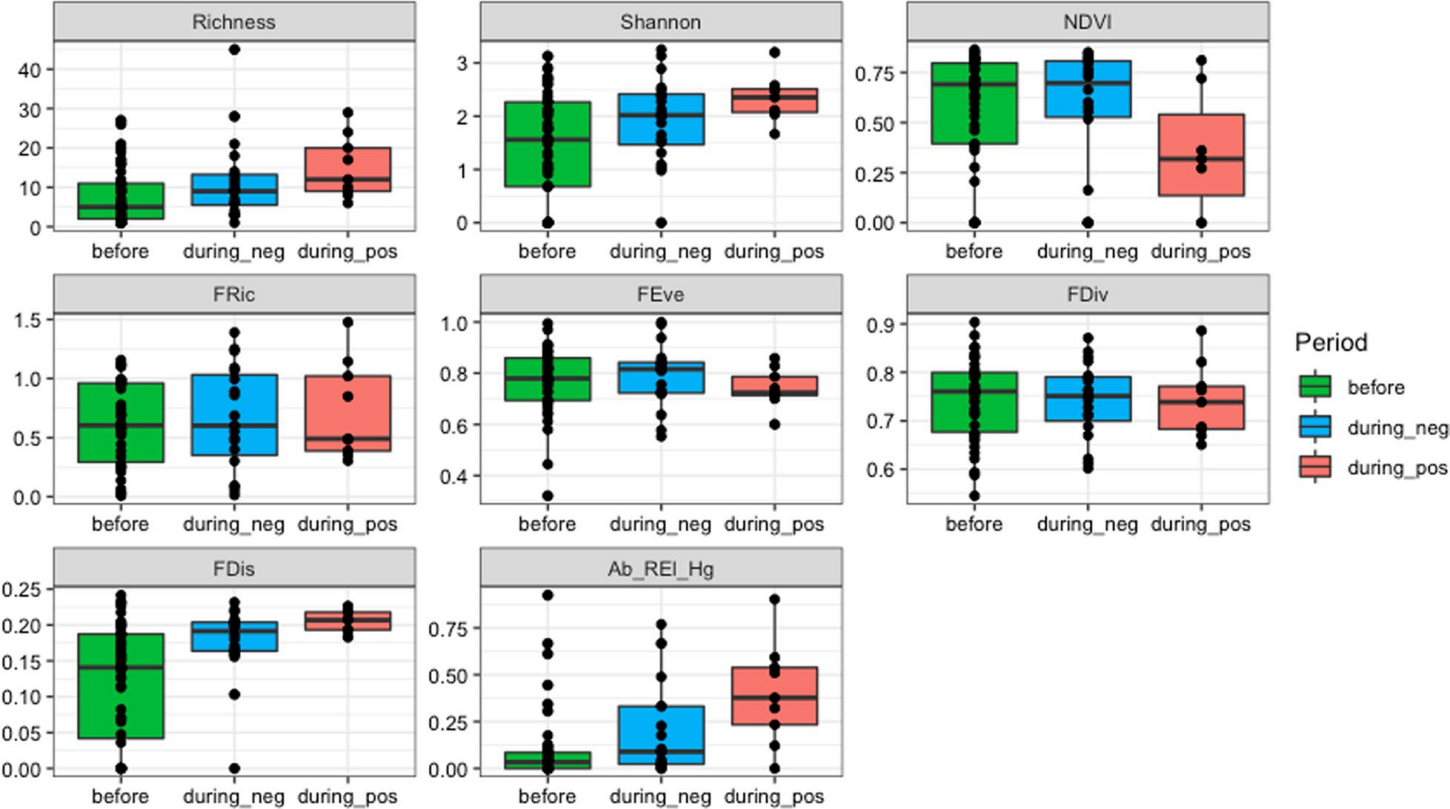
Biodiversity measures considering sampling points in three scenarios: before YFV outbreak vs. during YFV outbreak negative points vs. during YFV outbreak positive points

When comparing ecological aspects among the three epidemiological scenarios (before YFV outbreak, during YFV outbreak negative points, and during YFV outbreak positive points), it became clear that the mosquito biodiversity indicators (richness and Shannon–Wiener index) increased during the outbreak. Furthermore, sampling points with YFV-positive mosquitoes presented higher *Haemagogus* relative abundance and marginally higher FDis (*t* = −2.005, *df* = 25.499, *P* = 0.055). On the contrary, NDVI measurements were lower at sampling points with positive mosquitoes. The other biodiversity measures were not significantly different between the different scenarios (Fig. 3).

#### Comparisons between positive and negative mosquito sampling points during the yellow fever epidemic—infection predictors

For *Haemagogus* MIRs, model selection indicated that FDis, FRic, richness, and Shannon–Wiener index were the most important predictors, as they were included in all the top-ranked candidate models (proportion of explained deviance *R*^2^_*M*_ around 0.38 and relative importance of overall predictor = 1.00, Additional file 1: Tables S1 and S3). Model-averaged coefficients (based on a 95% confidence interval that excluded 0) for MIRs indicated that an increase in FDis and decrease in FRic, species richness, and Shannon-Weiner diversity led to an increase in the *Haemagogus* MIR (Table 3). FDis and relative abundance of *Haemagogus* were also important predictors of the positivity of sampling points, as they were included in most of the top-ranked candidate models (proportion of explained deviance *R*^2^_*M*_ around 0.20 and relative importance of overall predictor > 0.6, Additional file 1: Tables S2 and S4), but their model-averaged coefficients overlapped 0 (Table 4), evidencing a weak effect. Although included among the top-ranked candidate models, FDiv and relative abundance of *Haemagogus*, and species richness and FEve had a weak effect on *Haemagogus* MIRs and the positivity of sampling points, respectively (model-averaged coefficients for all covariates overlapped 0, Tables 3 and 4).

**Table 3:** Model-averaged standardized coefficients (based on models summarized in Additional file 1: Table S1), unconditional standard errors, 95% confidence intervals, and relative importance of biodiversity predictors of *Haemagogus* MIR in the sampling points during the 2017–2019 YFV outbreak in Rio de Janeiro, Brazil

	Standardized coefficient	Unconditional SE	95% CI		Relative importance of overall predictor
			2.50%	97.50%	
Intercept	−13.108	1.591	−16.331	−9.885	
FDis	112.523	8.945	94.242	130.815	1.00
FRic	−8.400	1.015	−10.489	−6.311	1.00
Richness	−1.558	0.341	−2.257	−0.860	1.00
Shannon	−0.972	0.274	−1.535	−0.409	1.00
FDiv	3.491	2.499	−1.474	8.458	0.71
Ab_Rel_Hg*	−0.282	0.468	−1.205	0.641	0.29

*Relative abundance of *Haemagogus*

**Table 4:** Model-averaged standardized coefficients (based on models summarized in Additional file 1: Table S2), unconditional standard errors, 95% confidence intervals, and relative importance of biodiversity predictors of the positivity of the sampling points during YFV outbreak in Rio de Janeiro, Brazil

	Standardized coefficient	Unconditional SE	95% CI		Relative importance of overall predictor
			2.50%	97.50%	
Intercept	−7.304	6.472	−20.433	5.825	
FDis	37.158	32.524	−28.6	102.916	0.80
Ab_Rel_Hg*	2.330	2.519	−2.732	7.392	0.65
Richness	−0.122	0.245	−0.615	0.371	0.35
FEve	−1.578	4.035	−9.646	6.490	0.20

*Relative abundance of *Haemagogus*

#### Comparisons over the entire sampled period

Among the tested geo-environmental factors (altitude, NDVI, fragment size, land use/land cover), NDVI was the only one that correlated with the abundance of *Hg. janthinomys*/*capricornii* (Pearson correlation *r* = 0.256, *P* = 0.02) and *Hg. leucocelaenus* (Pearson correlation *r* = 0.269, *P* = 0.014). Altitude, fragment size, and land use/land cover had no direct influence on the abundance of *Hg. janthinomys*/*capricornii* (Pearson correlations *r* = 0.017, *P* = 0.878; *r* = −0.007, *P* = 0.949; *r* = −0.175, *P* = 0.113, respectively) or *Hg. leucocelaenus* (Pearson correlations *r* = −0.177, *P* = 0.109; *r* = −0.001, *P* = 0.996; *r* = −0.215, *P* = 0.051, respectively).

### Geospatial analysis

**Fig. 4:**
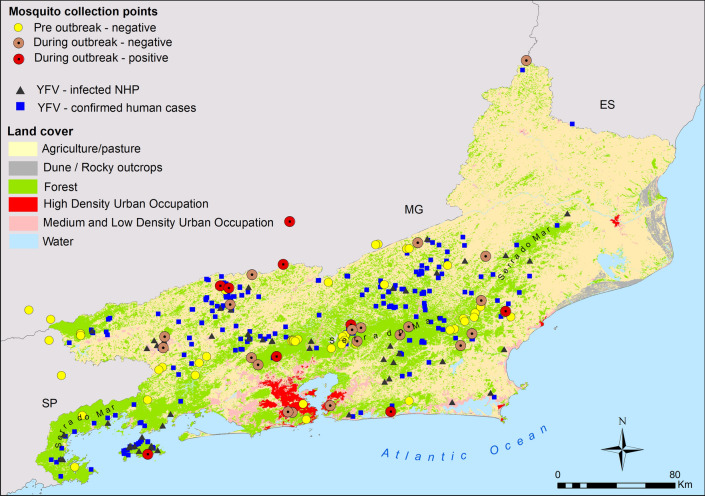
Rio de Janeiro state map showing land use/land cover along with mosquito sampling points and distribution of most probable places of infection for NHPs and humans infected with YFV

**Fig. 5:**
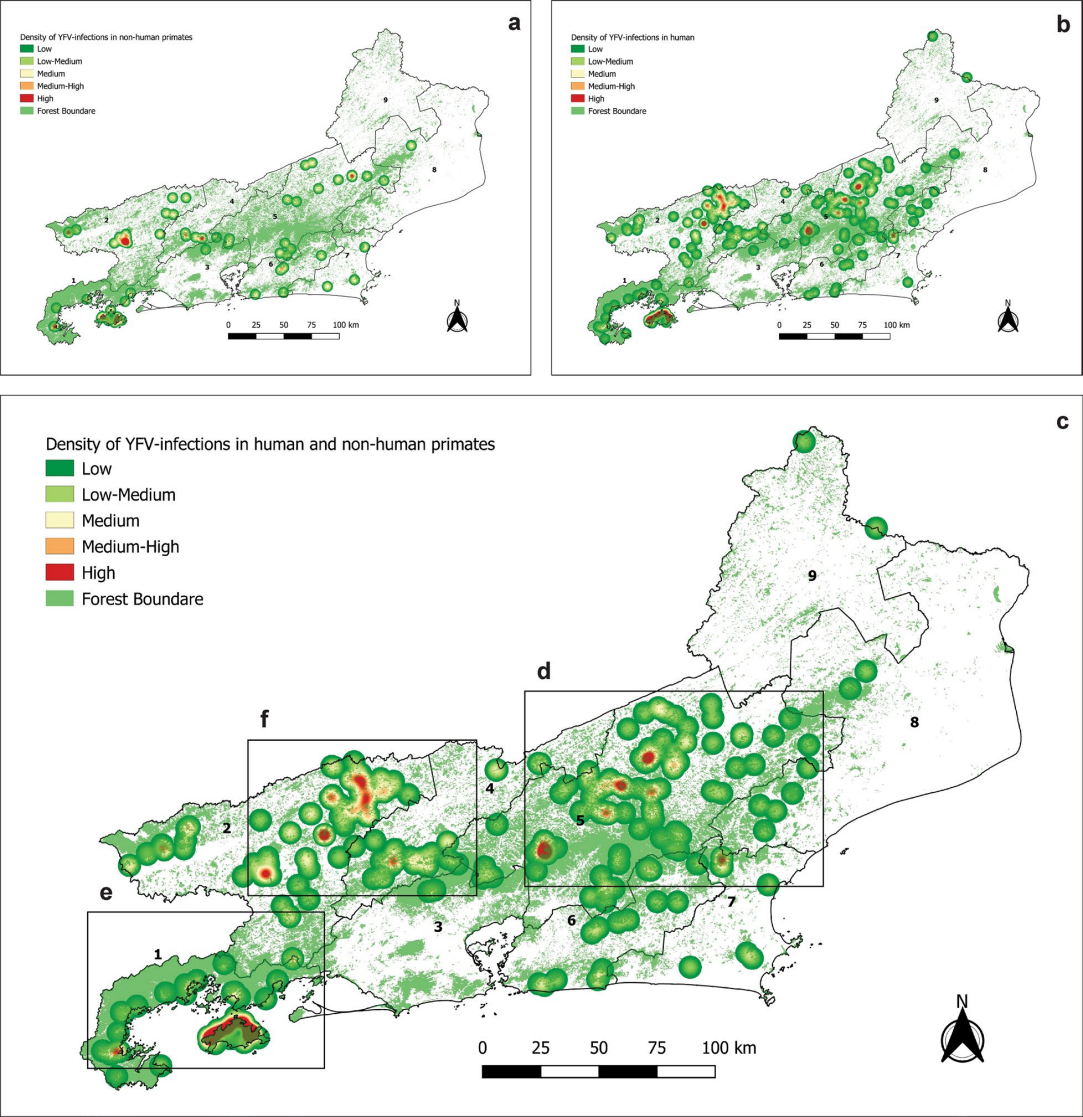
Kernel maps showing YFV infection density in Rio de Janeiro state, Brazil in 2015–2019. Areas covered by forests are also shown. **a** YFV infection density in NHP. **b** YFV infection density in humans. **c** YFV infection density in both NHP and humans. Rectangles **d**, **e** and **f** highlight three YFV infection clusters. Numbers indicate the regions of Rio de Janeiro: 1—Bahia da Ilha Grande, 2—Médio Paraíba, 3—Metropolitana I, 4—Centro-Sul, 5—Serrana, 6—Metroplotana II, 7—Baixada Litorânea, 8—Norte, 9—Noroeste

The geographical coordinates of the most probable places of YFV infection for 65 NHPs and 269 humans as well as the 81 mosquito sampling points in RJ were plotted on maps illustrating the predominant land use and vegetation cover throughout the state (Fig. 4). YFV-infected mosquitoes were found in six out of the 26 sampling points sampled during the yellow fever outbreak (Fig. 4). As expected, human and NHP YFV infections were abundant in areas with forest cover and forest fragments, but practically absent in areas predominantly covered by pastures and highly dense urbanized areas (Fig. 3). The kernel maps (Fig. 5) showed that, despite YFV infections in both NHPs (Fig. 5a) and humans (Fig. 5b) being distributed throughout almost half of the state, it is possible to identify at least three clusters with a greater number of records (Fig. 5c). Interestingly, two of these clusters (rectangles d and e) coincide with the most heavily forested areas, located in the Bahia da Ilha Grande and Serrana regions, and the third (rectangle f) is composed of small, highly fragmented forest, located in the Médio Paraíba and Centro-Sul regions (Fig. 4 and 5c).

**Fig. 6:**
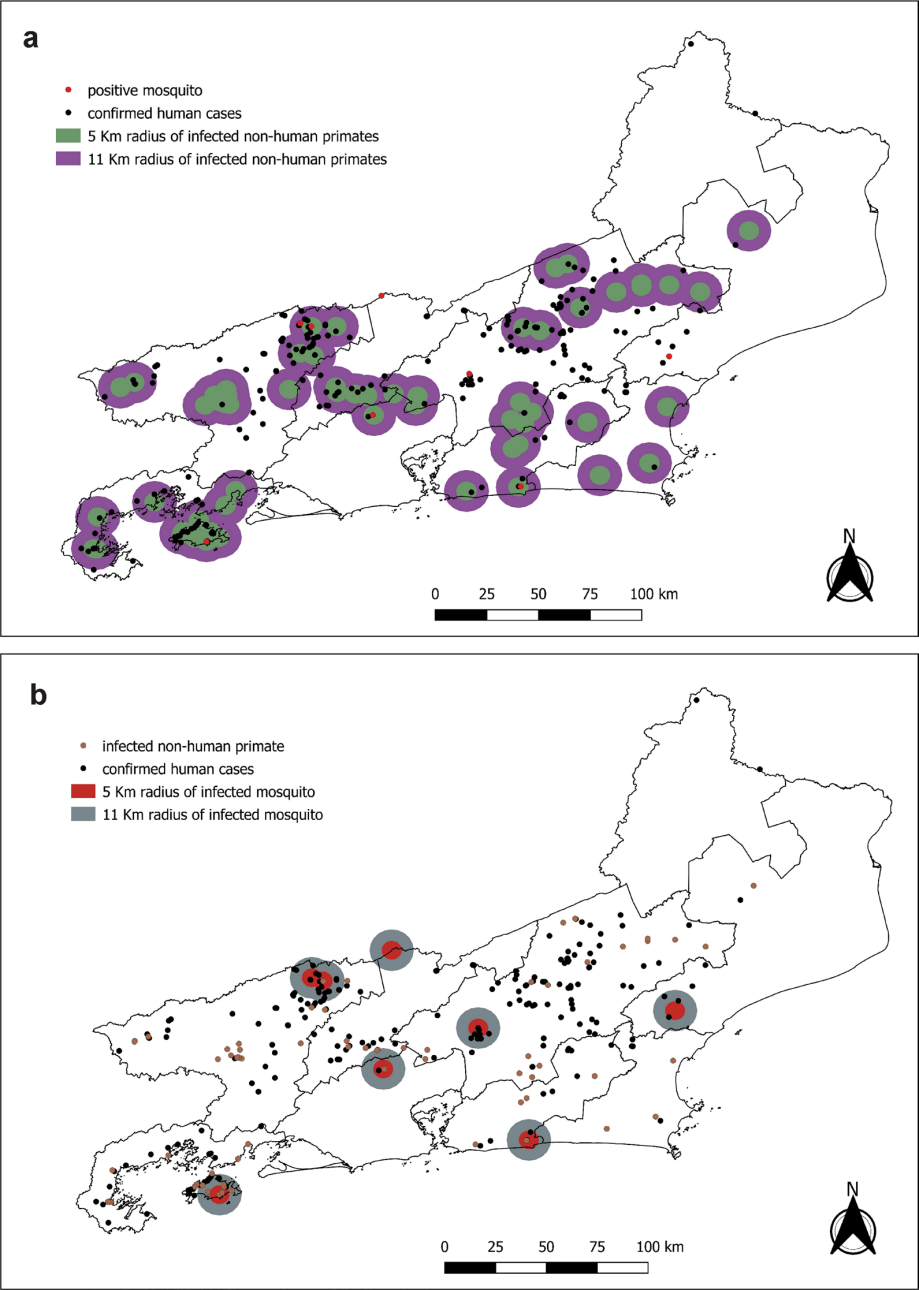
Maps showing 5- and 11-km radius from each point where an infected NHP (**a**) or mosquito (**b**) was found in Rio de Janeiro state, Brazil in 2015–2019. Confirmed human cases are shown as black dots

**Fig. 7:**
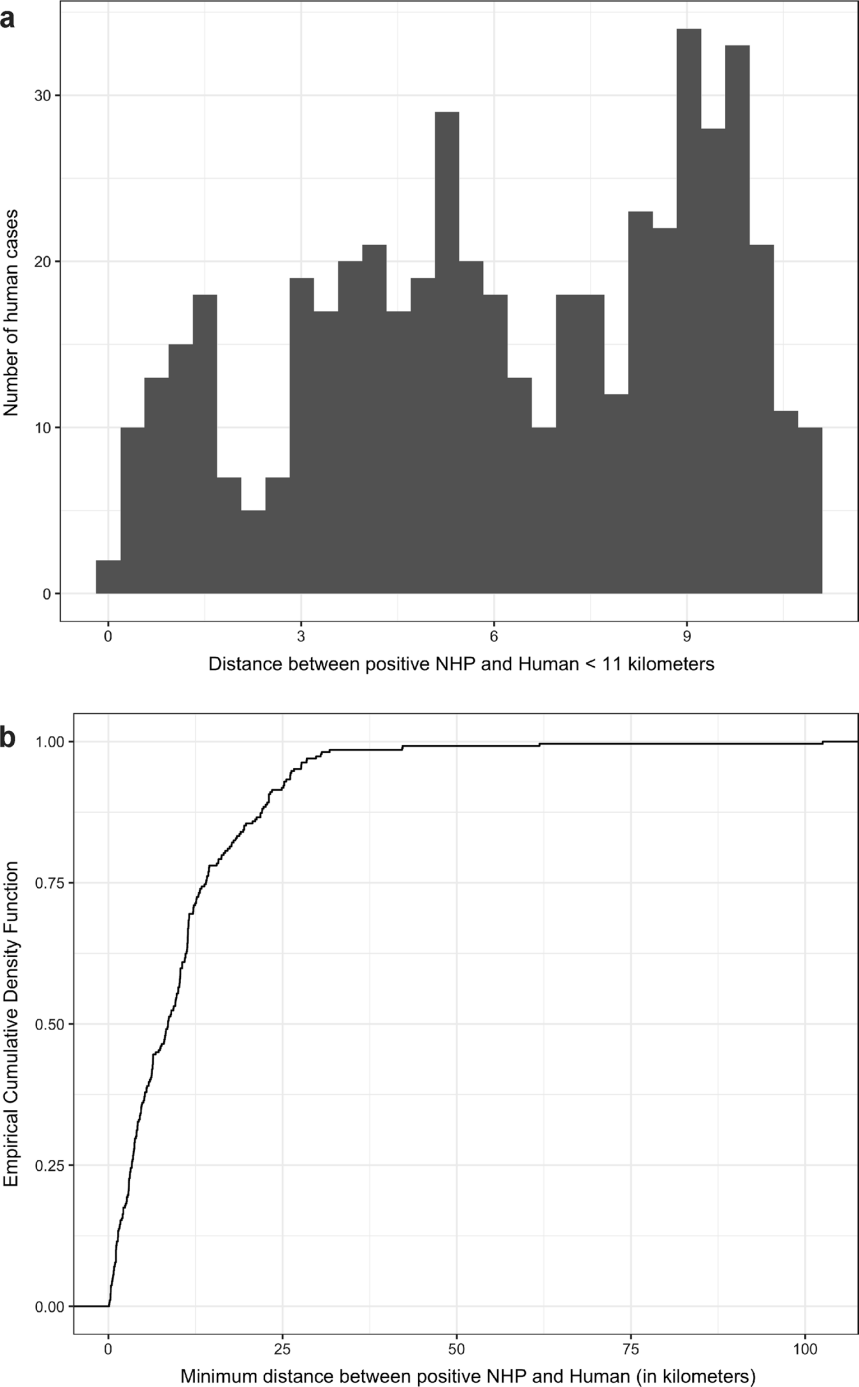
**a** Number of YFV human cases within a distance of 11 km from a YFV-infected NHP. **b** Cumulative percentage of YFV human cases as a function of the distance from a YFV-infected NHP in Rio de Janeiro state, Brazil in 2015–2019

We found that most of the YFV confirmed human cases were concentrated within radii of 5 and 11 km (compatible with the flight range of the primary vectors) around the PLI of YFV infection in NHPs (Fig. 6a) or in mosquitoes (Fig. 6b). In fact, 62% (*n* = 166) of the human cases were within a radius of 11 km of a YFV-positive NHP (Fig. 7a) and 90% were within 25 km (Fig. 7b).

## Discussion

Despite several studies investigating different aspects of the recent YFV outbreak in southeast Brazil, this is the first time that ecological and geographical analysis tools were used to shed light on the determinants of the spread and transmission strength of the virus in RJ, a state without YFV circulation for around 80 years. From the standpoint of mosquito biodiversity, we demonstrate that the increase in *Haemagogus* abundance and functional dispersion, together with the reduction in mosquito species richness and FEve associated with the increased abundance of functional group 1, seems to increase the risk of YFV infection in mosquitoes. From a geographical perspective, we show that YFV transmission was more intense in two different types of environments: in continuous forest areas and in mosaics composed of forest fragments surrounded by pastures. Furthermore, we show that the majority of PLI in humans are within a radius of 11 km from confirmed YFV epizootics in NHPs. Together, these data help us understand the complexity of the factors determining YFV dispersion and provide support for further prediction risk analyses and preventive measures which could protect human populations.

When we compared the three epidemiological scenarios (before YFV outbreak, during YFV outbreak positive points, and during YFV outbreak negative points), we found an increase in mosquito species richness, Shannon-Winner index, and *Haemagogus* abundance during the yellow fever outbreak. In other words, the increase in species richness offset the increase in *Haemagogus* abundance and increased the Shannon–Wiener index. Furthermore, the increased presence and abundance of the two species considered primary vectors of YFV in the southeastern outbreak (*Hg. janthinomys*/*capricornii* and *Hg. leucocelaenus*) [16, 43], combined with those of the potential vector *Ae. albopictus*, constituted the main differences between areas with and without detection of yellow fever in mosquitoes. Natural YFV infection has never been confirmed through viral isolation or complete genome sequencing in *Ae. albopictus* in Brazil, and Brazilian populations of this species have low vector competence for YFV [18, 44]. However, it can bite YFV-infected NHP on the forest ground layer [14], and YFV has the potential to adapt to this species [45], which is why it can be considered a potential vector [46]. *Haemagogus janthinomys*/*capricornii* and *Hg. leucocelaenus*, as well as *Ae. albopictus*, are tree-hole breeders, and lay eggs capable of resisting months of desiccation, hatching after one (for *Ae. albopictus*) or several (for *Haemagogus*) immersions in water [47–49]. Therefore, they are species that depend on and respond to similar environmental conditions. Concerning climate, their population peak is recorded in the rainy summer characterized by increased rainfall and temperature [24, 50], which also determines the seasonal period of yellow fever transmission in Brazil [51].

Regarding the functional diversity of mosquitoes, it is interesting to note that some characteristics are more abundant in forest environments (e.g. breeding sites in plant axils and in shallow pools on the ground, as well as solitary larvae breeders) and others in urban environments (e.g. artificial breeding sites). A greater number of traits are also found in forested areas, indicating greater diversity of ecological niches. Importantly, traits related to the biology of YFV vectors (e.g. vector competence, natural infection, skip oviposition) are striking among forest environments and in rural and urban forest fragments, which reflects the capacity of these environments to support populations of these vectors. Currently, the expansion of cities into forest fragments for estate speculation and/or the use of these fragments for leisure activities has been observed, which increases the chances of human infections in the sylvatic cycle and raises concerns regarding the risk of re-urbanization of yellow fever, especially in large cities surrounded by or interspersed with green spots [2]. São Paulo, Goiânia, and Nova Iguaçu are examples of large Brazilian cities with recent detection of yellow fever in their nearby forest areas, which increases the need for constant surveillance [6, 52, 53]. From the functional diversity analyses, it was possible to describe, for the first time, the existence of four main functional groups within the mosquito communities. The increased abundance of group 1, formed mainly by Aedini mosquitoes including *Haemagogus* species, primary vectors of yellow fever, in areas where positive mosquitoes were found may be an important indicator of areas receptive to viral circulation. Therefore, it will be important to carry out studies on the abundance of these functional groups to detect new areas receptive to YFV.

Curiously, although an increase in mosquito species richness and diversity was detected at the points sampled during the yellow fever outbreak, the *Haemagogus* MIRs showed the opposite behavior. That is, when comparing positive areas, we found that *Haemagogus* infection rates increased when mosquito diversity decreased and when relative abundance of *Haemagogus* increased, which could be explained by the concept of the “dilution effect.” The dilution effect describes the idea that the presence of different host species would increase the evenness of species abundance and dilute the chances of pathogen transmission, due to the presence of several incompetent reservoirs [54]. In this sense, in a more diverse region (greater number of vertebrate host and mosquito species), the probability of transmission of YFV is reduced due to greater availability of hosts—many of which are incapable of amplifying the virus—and competition for resources among vectors, which can be eclectic concerning blood-feeding sources [55]. Interestingly, we observed that the reduction in mosquito diversity, expressed by the Shannon–Wiener, richness, and FRic indices, positively influenced the *Haemagogus* MIR, corroborating the dilution effect theory. Similar results have been found for other mosquito-borne diseases. Decreasing bird and mosquito diversity, for example, has enhanced the incidence of human West Nile fever cases in the United States, and reduced functional bird diversity has increased the prevalence of avian malaria and the diversity of *Plasmodium* strains in the Brazilian Atlantic Forest [56–58]. In conclusion, the most important predictors of infection were the high relative abundance of *Haemagogus*, and consequently lower mosquito species richness and diversity. Areas and situations with these characteristics should be monitored by propensity for viral circulation. Nevertheless, we could not establish a *Haemagogus* population threshold that indicated major or imminent risk of YFV circulation in RJ, where the virus caused an outbreak and has disappeared since 2019. Cross-sectional studies in enzootic/endemic areas, such as the Amazon region, may help to unravel this issue.

The type of land cover/land use is highlighted as a determining factor for the occurrence of sylvatic yellow fever. Forested areas are more susceptible to viral circulation due to the higher probability of occurrence of NHP species and primary vector mosquito species that use water in tree holes for breeding [19, 21, 59, 60]. In fact, we found two important clusters of confirmed YFV infections in humans and NHPs in the areas most densely covered by forests, which in RJ correspond to the large forest continuum distributed along the mountain chain of Serra do Mar (Fig. 4 and 5c, rectangles d and e). However, a third cluster was verified in the Médio Paraíba and Centro-Sul regions of RJ (Fig. 5c, rectangle f). It is a mosaic composed of small forest fragments mostly surrounded by pastures (Fig. 5c, rectangle f). This type of landscape, characterized by intermediate levels of forest cover with numerous fragments producing higher availability of forest edges, was recently identified as more prone to the occurrence of human cases in a municipality-level analysis [61]. In this type of environment, the spread of the virus between the fragments possibly occurs through wind currents that facilitate and enhance the flight of vector mosquitoes [20, 30, 62]. Furthermore, humans become infected when approaching the fragments to rest, plant, harvest, or extract other forest resources. Recently, a correlation between the seasonality of agriculture and the appearance of YFV human cases in Brazil was demonstrated [63]. During planting and harvesting times, people working in agriculture and extractivism, who represent about 45% of the reported YFV cases, are more exposed to contact with the wild environment [63], which together with the dilution effect would help to explain the infection force in these regions with small forest fragments. Therefore, it appears that there are two distinct socio-ecological contexts, both favorable to the circulation of the YFV: The first is continuous forest environments that, due to the diversity and abundance of ecological niches, have a great support capacity for harboring many specimens of different species of NHP and of mosquitoes. In this context, humans would be exposed when entering the woods for tourism, leisure, or in search of bucolic moments nearby [2, 64], but would be partially protected by the dilution effect. The second is fragmented forest mosaics surrounded by pastures or plantations. Although small, these forest fragments can support groups of *Alouatta* and *Haemagogus* mosquitoes, the main vertebrate and invertebrate hosts, respectively. [16, 65–68]. In this context, humans, especially rural workers, would become infected when approaching the fragments during their work routine.

To the best of our knowledge, this is the first time that the minimum distance has been measured between the identification of a YFV-infected NHP and the occurrence of human infections. The 11-km radius, where 62% of human cases are concentrated, matches the flight capacity of the main vectors identified in RJ [16], nearly 6 km for *Hg. leucocelaenus* and 11 km for *Hg. janthinomys*/*capricornii* [30]. It is important to consider that finding a dead monkey and confirming it for YFV is not trivial, as it depends on the capacity of surveillance combined with the opportunity to collect viable samples for diagnosis [69]. The molecular analysis of a large set of YFV samples obtained from the 2017 and 2019 outbreak [9, 13, 70] has proved that the virus reached RJ from southern Espírito Santo. However, the large territorial gap in reports of YFV-infected NHPs between the southernmost border of Espírito Santo and the nearest NHP and human depicted in Fig. 3 highlights the failure of YFV epizootic surveillance. Therefore, it is possible that most of the YFV epizootics have not been detected and reported and/or laboratory-confirmed, which probably contributed to the increase in the distances measured. Even so, according to our data, strategic vaccination of the population within a radius of up to 25 km from a confirmed epizooty disease would prevent more than 90% of human cases, even in areas without a record of epizootic disease, considered epidemiological silent areas. Timely vaccination within a 2-km radius of a dead monkey helped to prevent several human cases in the 2008–2009 outbreak in southern Brazil [71]. The strategy of rapid and mobile vaccination campaigns to reach the more vulnerable rural populations within a radius of up to 25 km from the encounter of an infected mosquito may also be applied, especially when the surveillance of epizooties is failing. Although the opportunity for finding infected mosquito collections is even rarer, entomological and primatological surveys are complementary. In areas such as the city of Teresópolis, for example, where 22 human cases were confirmed and georeferenced, only positive mosquitoes were found, due to a lack of timely collection of NHP [15].

## Conclusions

Together, our results add new pieces to the puzzle towards understanding the epidemiology of sylvatic yellow fever in the Brazilian Atlantic Forest from data obtained prior to and during the outbreak in RJ. Importantly, we showed that there are at least two sets of favorable conditions for the circulation of yellow fever: areas with large forest continuums, capable of harboring many hosts of the virus, and areas with small forest fragments, where environmental imbalance can lead to a greater density of primary vectors, linking the loss of biodiversity as a risk factor. For both sets, changes in the relative abundance of functional groups and species composition of vector communities seem to determine not only the possibility of YFV circulation, but also the rate of vector infection and the occurrence of human cases. In this way, future analyses of vector biodiversity and landscape may help to predict areas permissive to yellow fever outbreaks.

## Supplementary Information

**Additional file 1: Table S1**. Top-ranked candidate models explaining variation in *Haemagogus* MIR in the sampling points during YFV outbreak in Rio de Janeiro, RJ, Brazil. **Table S2**. Top-ranked candidate models explaining variation in the positivity of sampling points during YFV outbreak in Rio de Janeiro, RJ, Brazil.

## Data Availability

The datasets generated and/or analyzed during the current study are available from the corresponding author on reasonable request.
