# Supplementary material for: Ecological and environmental factors affecting transmission of sylvatic yellow fever in the 2017–2019 outbreak in the Atlantic Forest, Brazil
Source: Parasit Vectors. 2022 Jan 10;15:23. doi: 10.1186/s13071-021-05143-0 (PMC8750868; doi:10.1186/s13071-021-05143-0)
Supplement: Supplementary file 1 — Additional file 1: Table S1. Top-ranked candidate models explaining variation in Haemagogus MIR in the sampling points during YFV outbreak in Rio de Janeiro, RJ, Brazil. Table S2. Top-ranked candidate models explaining variation in the positivity of sampling points during YFV outbreak in Rio de Janeiro, RJ, Brazil. [file 13071_2021_5143_MOESM1_ESM.docx]

S1 Table. Top-ranked candidate models explaining variation in *Haemagogus* MIR in the sampling points during YFV outbreat at Rio de Janeiro, RJ, Brazil. AICc = Akaike Information Criteria corrected for small sample sizes. R2M = McFadden index.

| Model | df | Log-likelihood ratio | AICc | ∆AICc | AICc weight | R2M |
| --- | --- | --- | --- | --- | --- | --- |
| FDis+FDiv+FRic+Richness+Shannon | 8 | -36.33 | 96.2 | 0.00 | 0.60 | 0.38 |
| Ab_Rel_Hg+FDis+FRic+Richness+Shannon | 8 | -37.23 | 98.0 | 1.8 | 0.25 | 0.37 |

S2 Table. Top-ranked candidate models explaining variation in the positivity of sampling points during YFV outbreat at Rio de Janeiro, RJ, Brazil. AICc = Akaike Information Criteria corrected for small sample sizes. R2M = McFadden index.

| Model | df | Log-likelihood ratio | AICc | ∆AICc | AICc weight | R2M |
| --- | --- | --- | --- | --- | --- | --- |
| Ab_Rel_Hg+FDis | 3 | -14.14 | 35.3 | 0.00 | 0.229 | 0.20 |
| Ab_Rel_Hg+FDis+FEve | 4 | -12.92 | 35.6 | 0.31 | 0.196 | 0.26 |
| FDis+Richness | 3 | -14.60 | 36.2 | 0.91 | 0.145 | 0.17 |
| FDis | 2 | -16.04 | 36.6 | 1.29 | 0.120 | 0.09 |
| Ab_Rel_Hg+FDis+Richness | 4 | -13.476 | 36.7 | 1.42 | 0.113 | 0.23 |
| Ab_Rel_Hg | 2 | -16.14 | 36.8 | 1.48 | 0.109 | 0.08 |
| Richness | 2 | -16.35 | 37.2 | 1.90 | 0.088 | 0.07 |
